# Disease progression in dementia with Lewy bodies: A longitudinal study on clinical symptoms, quality of life and functional impairment

**DOI:** 10.1002/gps.5839

**Published:** 2022-11-20

**Authors:** Marleen van de Beek, Annemartijn van Unnik, Inger van Steenoven, Jessica van der Zande, Frederik Barkhof, Charlotte E. Teunissen, Wiesje van der Flier, Afina W. Lemstra

**Affiliations:** ^1^ Department of Neurology Alzheimer Center Amsterdam Amsterdam Neuroscience Vrije Universiteit Amsterdam Amsterdam UMC Amsterdam The Netherlands; ^2^ Department of Radiology and Nuclear Medicine Amsterdam UMC Amsterdam The Netherlands; ^3^ Institutes of Neurology and Healthcare Engineering UCL London England UK; ^4^ Department of Clinical Chemistry Neurochemistry Laboratory Amsterdam Neuroscience Vrije Universiteit Amsterdam Amsterdam UMC Amsterdam The Netherlands; ^5^ Department of Epidemiology and Data Sciences Vrije Universiteit Amsterdam Amsterdam UMC Amsterdam The Netherlands

**Keywords:** dementia with Lewy bodies, IADL, progression, quality of life

## Abstract

**Background and Objectives:**

Dementia with Lewy Bodies (DLB) is a heterogeneous disease, with variable signs and symptoms across multiple domains. We aimed to identify associations with rate of change in cognition, everyday functioning (IADL) and quality of life (QoL).

**Methods:**

We included 121 DLB patients (69 ± 6 yrs, 14%F, MMSE: 25 ± 3) in our prospective cohort (follow‐up 2 ± 1 yrs). We described progression of symptoms and cognitive decline over time. Mixed models were used to investigate whether changes in symptoms were associated to changes in IADL (FAQ), QoL (QoL‐AD) and caregiver burden (ZBI). Last, we investigated whether baseline symptoms and biomarkers predicted decline in cognition (MMSE), IADL (FAQ) and QoL (QoL‐AD).

**Results:**

Parkinsonism and RBD were most frequently present early in the disease course, while hallucinations were more likely to develop in a later stage. MMSE (annual change *β* ± SE = −2.06 ± 0.23), QoL‐AD (−1.03 ± 0.20), and FAQ (3.04 ± 0.30) declined over time. Increasing severity of clinical symptoms was associated to increases in FAQ, QoL‐AD and caregiver burden. Baseline clinical symptoms were not predictive for decline in these outcomes. By contrast, AD co‐pathology (CSF pTau/Aβ42 ratio) was associated to steeper decline in MMSE (−1.23 ± 0.54). Medial temporal atrophy (−0.81 ± 0.26) and global cortical atrophy (−0.73 ± 0.36) predisposed for decline in QoL‐AD.

**Conclusions:**

Our findings imply that underlying disease processes, rather than clinical symptomatology aid in predicting decline. These findings are relevant for treatment strategies and the development of DLB specific outcome measures.

## Key points

- Clinical symptoms did not determine progression of cognitive functioning, IADL or quality of life (QoL).
- Concomitant AD pathology and atrophy were determinants for progression in dementia with Lewy bodies.
- Increasing severity of clinical symptoms was associated with cognitive decline and diminished QoL and functional abilities.

## 1 INTRODUCTION

Patients with dementia with Lewy Bodies (DLB) have a highly variable presentation of symptoms. The cognitive profile of DLB is characterized by impairment in attention, executive and visuospatial functions, and fluctuations. Besides cognitive impairment, patients may experience neuropsychiatric symptoms, such as visual hallucinations, delusions, apathy, and depressive symptoms. Other DLB features include physical symptoms, namely parkinsonism, RBD and autonomic dysfunction.^1^ Different symptoms have different effects on disease burden, as neuropsychiatric symptoms are associated with reduced quality of life (QoL), while cognitive and physical symptoms have been related to impairment in daily living (IADL).^2^, ^3^

DLB has an unfavorable prognosis when compared to Alzheimer's disease (AD). Patients with DLB have more rapid cognitive decline, higher mortality risk and earlier nursing home admission.^4^, ^5^, ^6^, ^7^ Former studies estimating disease trajectories in DLB were often limited to estimating global cognitive decline, while it is debatable whether this is the most relevant measure of disease burden from the patients' perspective. When providing treatment, the ultimate goal is to optimize patients' QoL and limit impairment in daily living. As such, it is essential to gain insight into how progression of symptoms affects these outcomes. Increased understanding of the contribution of symptoms to disease burden could give targets for symptomatic treatment.

In the present study, we described the progression in symptoms and cognitive domains over time. We aimed to assess how changes in symptoms are associated with changes in functional outcomes and QoL. Last, we aimed to identify factors that influence progression in functional outcomes (cognition, IADL and QoL).

## 2 METHODS

We included 121 patients with DLB (*n* = 91)^1^ and MCI due to Lewy Bodies (MCI‐LB) (*n* = 30)^8^ from the DEvELOP cohort,^2^ embedded within the Amsterdam Dementia cohort.^9^ In short, patients were referred to Alzheimer Center Amsterdam and underwent diagnostic screening for dementia that included neurological, physical and neuropsychological evaluation, brain imaging, laboratory work and lumbar puncture.^9^ Diagnoses were made in a multidisciplinary meeting. In the case of DLB or MCI‐LB diagnosis, patients were invited to participate in DEvELOP. Exclusion criteria were severe physical or life‐threatening conditions and nursing home admittance. All patients gave written informed consent for use of their clinical data. The local medical ethics committee approved the study.

### 2.1 Clinical follow‐up

The first patient was included in 2016 and follow‐up is still ongoing. Patients were invited for annual follow‐up, during which the clinical workup was repeated. All patients were invited for at least one 1 year follow‐up and 92% of the patients that were still available for follow‐up had 2 follow‐up visits. For MCI‐LB patients, their diagnosis was re‐evaluated during these follow‐up visits. Progression to dementia was defined as having two or more impaired cognitive domains at neuropsychological assessment, accompanied by interference in daily living.

### 2.2 Clinical measures

Within DEvELOP, several questionnaires were administered to evaluate core and suggestive symptoms, IADL and QoL. The operationalization of symptoms and outcomes used in this study are summarized in Table 1. Duration of complaints was assessed during the patients' first visit and was operationalized as the time between diagnosis and the moment when the patient first noticed their cognitive complaints. Parkinsonism was assessed with the Movement disorders society Unified Parkinson's disease rating scale—UPDRS III (motor subscale).^10^ Parkinsonism was rated as present when the patient had bradykinesia (UPDRS‐bradykinesia≥1) with additional rigidity (≥1) and/or resting tremor (≥1). Subscales of the Neuropsychiatric Inventory (NPI) were used to assess presence of hallucinations, delusions, anxiety and apathy (cutoff per subscale ≥1).^11^ Presence of fluctuations was assessed using the Mayo Fluctuations questionnaire (MFQ, cutoff ≥3).^12^ RBD was assessed with the Mayo Sleep questionnaire (MSQ, cutoff ≥1). We used the Geriatric Depression Scale (GDS) 15‐items as a measure of depressive symptoms.^13^ Severity of orthostatic hypotension was defined by the difference in systolic blood pressure between lying and 3 min after standing. Constipation and urinary problems were assessed with the Non Motor Symptoms Scale (NMSS)^14^ or with the SCOPA‐AUT^15^ (*n* = 22). We dichotomized presence of symptoms with the NMSS questions 21 and 22 SCOPA‐AUT questions 5 and 8.

**TABLE 1:** Baseline characteristics and annual change in symptoms

	*N* = 121	
Sex, *n* female (%)	17 (14%)	
Age, years	69 ± 6	
Duration of complaints, years	3 [2‐5]	
Syndrome diagnosis, MCI/dementia	30/91 (25%/75%)	
Education, years	12 ± 3	
Follow‐up duration, years	2 ± 1	
Loss to follow‐up, *n* (%)	55 (45%)	
Time to loss to follow‐up	2.0 ± 0.9	
Core clinical symptoms		Annual change
MMSE	25 ± 3	−2.06 ± 0.23**
Hallucinations, (NPI, frequency*severity, *n* = 109)	1.39 ± 2.68	0.44 ± 0.16*
Hallucinations, *n* present (%)	43 (38%)	
Parkinsonism (UPDRS, *n* = 118)	21 ± 12	5.37 ± 0.55*
Fluctuations (MFQ, *n* = 116)	2 [1‐3]	0.15 ± 0.05
RBD (MSQ, *n* = 117)	87 (74%)	
OH (drop SBP, *n* = 119)	20 ± 21	
Urinary (NMSS/SCOPA‐AUT, *n* = 119)	59 (49%)	
Constipation (NMSS/SCOPA‐AUT, *n* = 119)	44 (37%)	
Delusions, (NPI, frequency*severity, *n* = 109)	0.56 ± 2.01	0.33 ± 0.11*
Delusions, *n* present (%)	13 (11%)	
Apathy (NPI, frequency*severity, *n* = 109)	2.89 ± 3.14	0.03 ± 0.14
Apathy, *n* present (%)	69 (60%)	
Anxiety (NPI, frequency*severity, *n* = 109)	1.43 ± 2.16	0.32 ± 0.14*
Anxiety, *n* present (%)	52 (45%)	
Depressive symptoms (GDS, *n* = 115)	4 ± 3	0.24 ± 0.11*
Disease burden		
IADL (FAQ)	12 ± 6	3.04 ± 0.30*
Quality of life (QoL‐AD)	31 ± 5	−1.03 ± 0.20*
Caregiver burden (ZBI)	24 ± 15	3.44 ± 0.57*
Biological measurements		
CSF pTau/Aβ42 ratio abnormal (*n* = 83)	48 (58%)	
DAT‐scan, abnormal (*n* = 95)	83 (87%)	
APOE‐e4 carrier (*n* = 90)	45 (50%)	
MTA, median [IQR]	1 [0–2]	
Global cortical atrophy, median [IQR]	1 [0–1]	
EEG abnormal, *n* (%)	78 (96%)	

*Note*: Data represent mean ± SD, n (%) or median [IQR]. Annual change in scores was estimated with linear mixed models, data represent β ± SE *p < 0.05, **p < 0.001.

Abbreviations: FAQ, Functional activities questionnaire; GDS, Geriatric depression scale; MFQ, Mayo Fluctuations Questionnaire; MMSE, Mini‐Mental State Examination; MSQ, Mayo Sleep Questionnaire; NMSS, non motor symptoms scale; NPI, neuropsychiatric inventory; QoL‐AD, Quality of Life—Alzheimer's disease; SBP, systolic blood pressure; UPDRS, Unified Parkinson's disease rating scale.

IADL interference was measured with the Functional activities questionnaire (FAQ, range 0–30). Higher scores indicate higher interference and a score higher than 7 is indicative of cognitive impairment.^16^, ^17^ QoL measured with the Quality of Life‐AD (QoL‐AD) questionnaire (range 0–30) with higher scores indicating higher QoL.^18^ Caregiver burden was measured with the Zarit burden interview (ZBI, range 0–88). Scores higher than 21 indicate mild to moderate caregiver burden, and high.^19^

### 2.3 Neuropsychological assessment

At baseline and follow‐up, patients underwent extensive neuropsychological assessment. We used MMSE as a measure of global cognition. Attention was assessed using the Trail‐Making Test‐A (TMT‐A), Stroop‐I and Stroop‐II and digit span‐forward.^20^, ^21^, ^22^ Memory functioning was measured with the Dutch version of the Rey Auditory Verbal Learning test (RAVLT, immediate and delayed recall), the Visual Association Test version A (VAT‐A) and the 3 min delayed recall of the Rey complex figure test.^23^, ^24^, ^25^ Executive functioning was measured with TMT‐B (ratio TMT‐B/TMT‐A), Stroop‐III (ratio Stroop‐III/Stroop‐II), digit span backwards (corrected for digit span forward), the Frontal Assessment Battery and letter fluency tests.^21^, ^22^ Visuospatial functioning was assessed using three subtests of the Visual Object and Space Perception battery (number location, fragmented letters, dot counting) and the copy of the Rey complex figure^25^, ^26^ Language was assessed with animal fluency and VAT‐naming subtest.^24^, ^27^ Time dependent tests were inverted so that higher scores reflect better performance. We calculated z‐scores using the baseline data as a reference. Next, we created composite scores by calculating the average Z‐score of each test in the domain. Composite scores were only calculated if at least two tests in the corresponding domain were available.

### 2.4 Baseline biological measures

MRI scanning was performed according to the standardized dementia protocol and visual assessment of atrophy was performed by experienced neuroradiologists.^9^ Medial temporal lobe atrophy (MTA) was rated using coronal T1‐weighted images on a 5‐point scale (0–4).^28^ Global cortical atrophy (GCA) was rated on axial images using a 4‐point scale (0–3).^29^ Baseline MRI was available for *n* = 109 (90%) patients.

CSF Aβ42, Tau and pTau concentrations were measured using a sandwich ELISA (Innotest, Fujirebio), or the Elecsys Aβ42, Tau, and pTau (181P) CSF assays run on the *cobas e*601 analyzer (Roche Diagnostics GmbH). Concomitant AD pathology was defined as a ratio of pTau/Aβ42 > 0.054 (Innotest) or pTau/Aβ42 > 0.020 (Elecsys).^30^ Baseline CSF was available for *n* = 83 (69%) patients.

Dopamine transporter imaging (^123^FP‐CIT(DAT)‐SPECT) was performed on discretion of the clinician to confirm diagnosis and was available for *n* = 95 (79%) patients. The SPECT imaging protocol has been described in detail in a previous report.^31^ Visual assessments and age‐matched BRs were taken into account in determining whether the scan was normal or abnormal.^31^

EEGs were recorded as standard screening of the memory clinic using a digital EEG system and software (Brain RT®; OSG b.v.). The EEG registrations were visually assessed by certified neurophysiologists and were scored according to a standardized visual rating scheme.^32^ From this scheme, the 5 point scale to assess the severity of EEG abnormalities was used, in which a severity score of higher than 1 indicated an abnormal EEG. EEG was available for *n* = 81 (67%) patients.

### 2.5 Statistical analyses

Analyses were performed in Rstudio 4.0.3. First, we used descriptive statistics to describe baseline characteristics and the longitudinal aspects of our cohort. For longitudinal analyses, we visualized the occurrence and likelihood of developing core clinical symptoms over time using cox proportional hazards analyses. We used the first occurrence of the symptom as an event and used the time between the baseline visit and the visit of the first occurrence as time. Longitudinal changes in severity of symptoms, cognition, IADL, QoL and caregiver burden were assessed with linear mixed models analyses (LMM). We created separate models per outcome of interest (dependent variable). These models included variables of interest as dependent variables, with time added as a fixed factor and random factors for individual intercept and time.

Next, to investigate how changes in symptoms related to changes in outcomes, we calculated difference scores (delta (*Δ*) scores) of symptom scores and outcomes between visits (*Δ* symptom = ‘symptom score at t’, minus ‘symptom score at *t*‐1’). We standardized these deltascores to make comparisons between predictors possible. Standardization was performed by making *Z*‐scores of delta‐scores ((delta score—mean delta score)/SD). LMMs were performed, accounting for repeated measures, with *Δ* symptoms as fixed factors and Δ outcome (ΔFAQ, ΔQoL, ΔZBI) as dependent variables and included a random intercept.

To identify which baseline measures were associated to decline in cognition, IADL and QoL over time, we applied LMMs with an interaction between time and the potential baseline determinants. We performed separate models for clinical symptoms (UPDRS‐III motor score, NPI‐hallucinations, MFQ, GDS, MSQ), and biological determinants (CSF AD profile (yes/no), EEG (abnormal yes/no), DAT‐SPECT (abnormal yes/no), MTA, GCA). Models included time, age and sex as fixed factors, and included a random intercept and random slope for time. *p*‐values were corrected for multiple testing with the FDR method. Significance was set at *p* < 0.05.

## 3 RESULTS

At baseline, participants were 69 ± 6 years old, 14% were female and MMSE was 25 ± 3. Mean duration of follow‐up was 2 ± 1 years (Table 1). Of 26 MCI‐LB patients with at least one follow‐up visit, 15 (58%) progressed to dementia after 1.5 ± 0.9 years. Of 121 patients, *n* = 14 patients completed the 4‐year follow‐up and for *n* = 53 patients follow‐up is still ongoing. Reasons for loss‐to‐follow‐up were death (*n* = 16, mean time to death 1.7 ± 0.9 years), nursing home admission (NAH) (*n* = 21, mean time to NAH 2.5 ± 0.8 years) or disease burden (*n* = 17, mean time to loss to follow‐up 1.8 ± 1.0 years).

### 3.1 Longitudinal symptoms, functional outcomes and cognition

**FIGURE 1:**
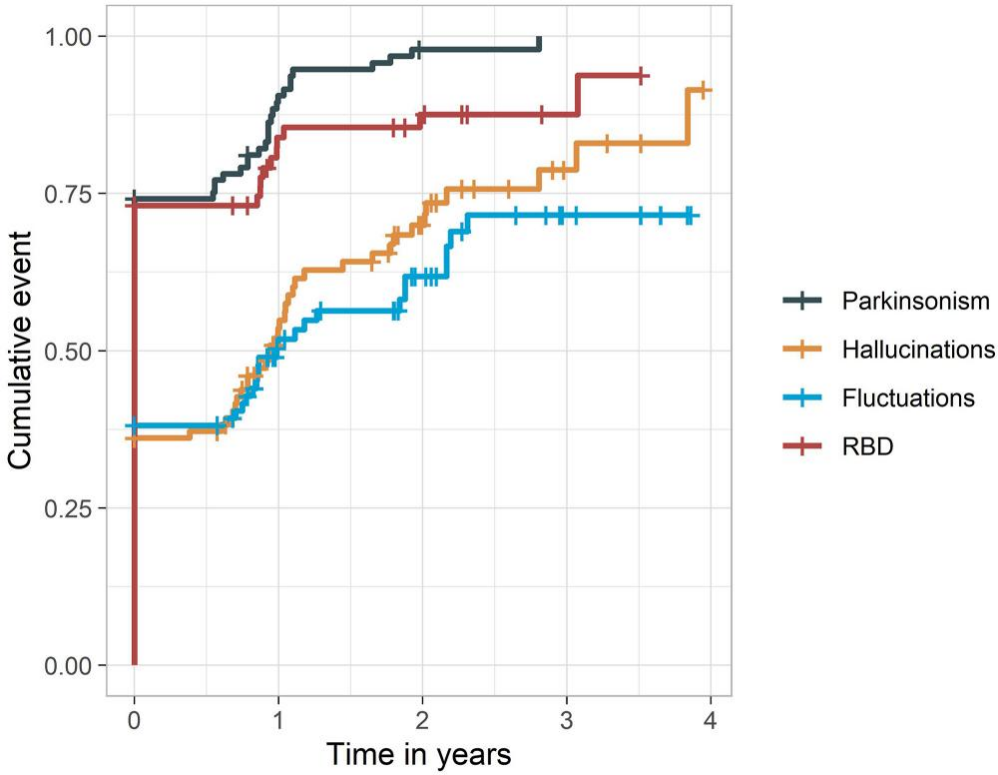
Cumulative likelihood of the presence of core clinical features during the course of the follow‐up, as calculated with cox‐proportional hazards. RBD, rapid eye movement sleep behavior disorder.

At baseline, mean UPDRS‐III was 21 ± 12 and 75% of patients fulfilled criteria for parkinsonism (bradykinesia with additional tremor and/or rigidity). The severity of parkinsonism increased over time (annual change UPDRS‐III: *β* ± SE = 5.37 ± 0.55) and the likelihood of having parkinsonism after 2 years follow‐up was nearly 100% (Figure 1). NPI‐hallucinations was indicative for presence of hallucinations in 38% patients at baseline. The NPI‐hallucinations score increased over time (annual change NPI‐hallucinations: *β* ± SE = 0.44 ± 0.16). The cumulative likelihood of having hallucinations was over 75% after 3 years follow‐up. Median MFQ was 2 [IQR 1–3], 39% of patients had an MFQ indicative of presence of fluctuations. The likelihood of having fluctuations was lowest of all symptoms and remained at 70% after 2 years follow‐up. RBD. Self‐reported or caregiver‐reported RBD symptoms were present in 74% of patients, this slightly increased over time, up to 82%.

For suggestive symptoms, we found that at baseline, nearly half of the patients (49%) had urinary problems and 37% had signs of constipation. Suggestive neuropsychiatric symptoms were common, 60% of the caregivers reported apathy (NPI frequency * severity: 2.9 ± 3.1), 45% reported anxiety (NPI frequency * severity: 1.4 ± 2.6),. Delusions were present in 11% of patients (NPI frequency * severity: 0.6 ± 2.0). Mean GDS was 4 ± 3. Over time, severity and frequency increased for delusions (annual change *β* ± SE = 0.33 ± 0.11), anxiety (*β* ± SE = 0.32 ± 0.14) and depressive symptoms (*β* ± SE = 0.24 ± 0.11). Apathy scores did not show a significant increase.

### 3.2 Longitudinal functional outcomes and cognition

**FIGURE 2:**
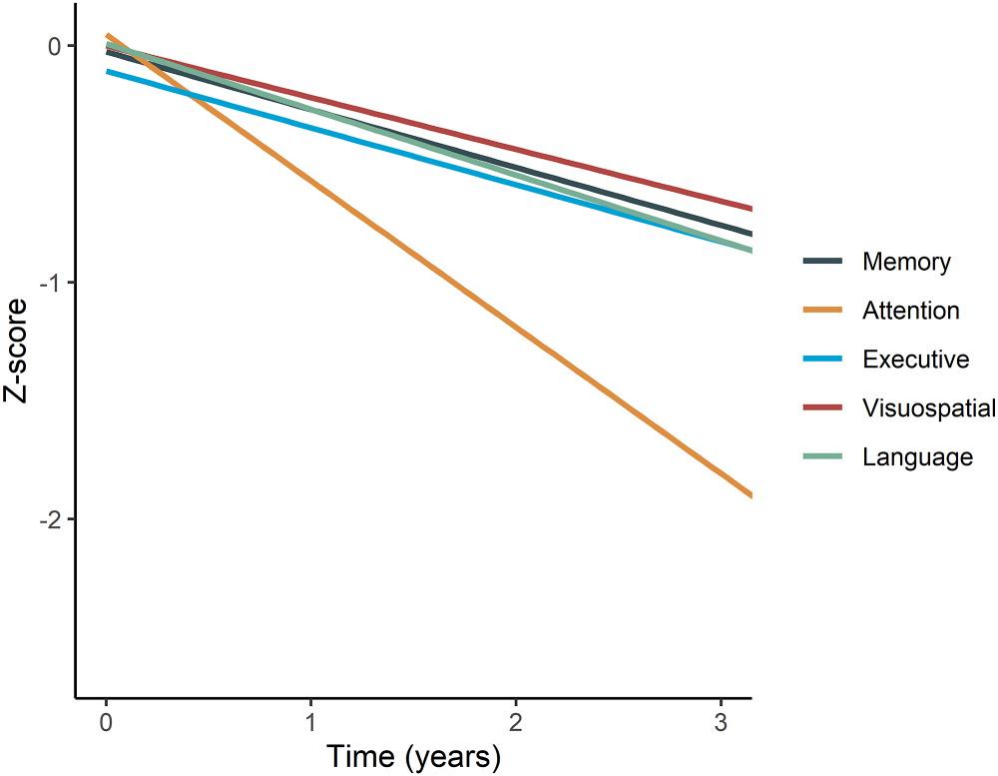
Cognitive domains over time. Data are *Z*‐transformed using baseline values as reference. Slopes are estimated with linear mixed models, corrected for age and sex.

IADL dependency increased during follow‐up, with an increase of 3.04 ± 0.30 points per year on the FAQ. Likewise, we observed a decrease in QoL over time (*β* ± SE = −1.03 ± 0.19). Caregiver burden increased over time, with 3.43 ± 0.58 annual increase on the ZBI. MMSE declined over time (annual change: *β* ± SE = −2.06 ± 0.23). When comparing different cognitive domains, we found that all cognitive domains declined over time (Figure 2). The steepest decline was observed in the attention domain (*β* + SE = −0.62 ± 0.07). On individual tests, TMT‐A (*β* + SE = −0.79 ± 0.10) and Stroop‐II (*β* + SE = −0.84 ± 0.12) showed steepest decline.

### 3.3 Changes in symptoms relate to changes in IADL and QoL

We applied LMM on repeated delta scores per individual to assess whether changes in symptoms were related to changes in functional outcomes (Table 2). Decline in MMSE and increases in parkinsonism, visual hallucinations, fluctuations and orthostatic hypotension were associated to a stronger increase in IADL dependency, of which fluctuations showed strongest association. We found that changes in depressive symptoms paired with decline in QoL, but this association did not survive FDR correction. Increases in fluctuations and hallucinations were associated to increases in caregiver burden, the latter association did not survive FDR correction.

**TABLE 2:** Association of changes in symptoms to changes in IADL, quality of life (QoL) and caregiver burden

	*Δ* FAQ	*Δ* QoL‐AD	*Δ* ZBI
*Δ* Cognition (MMSE)	−0.19 ± 0.08**	0.14 ± 0.08	−0.15 ± 0.09
*Δ* Parkinsonism (UPDRS)	0.18 ± 0.08**	−0.08 ± 0.07	0.10 ± 0.09
*Δ* Hallucinations (NPI)	0.23 ± 0.08**	−0.11 ± 0.08	0.16 ± 0.08*
*Δ* Fluctuations (MFQ)	0.33 ± 0.07**	−0.12 ± 0.08	0.24 ± 0.08**
*Δ* RBD (MSQ)	0.02 ± 0.07	8.81 ± 12.29	−0.08 ± 0.07
*Δ* Orthostatic hypotension (yes/no)	0.17 ± 0.08*	−0.12 ± 0.08	−0.05 ± 0.09
*Δ* Depressive symptoms (GDS)	0.05 ± 0.08	−0.18 ± 0.08*	−0.09 ± 0.08

*Note*: Data represent *β* ± SE as estimated by linear mixed models, using difference (*Δ*) scores between follow‐up moments for outcomes and predictors per individual. To enable comparison, difference scores were standardized before analyses. Bold depicts significant associations. *p < 0.05, **p < 0.5 after FDR correction.

Abbreviations: FAQ, Functional activities questionnaire; GDS, Geriatric depression scale; MFQ, Mayo Fluctuations Questionnaire; MSQ, Mayo Sleep Questionnaire; NPI, neuropsychiatric inventory; QoL‐AD, Quality of Life—Alzheimer's disease; SBP, systolic blood pressure; UPDRS, Unified Parkinson's disease rating scale; ZBI, Zarit burden interview.

### 3.4 Factors that influence disease progression

Table 3 displays the estimated association between baseline symptomatology and biological measures on baseline cognition (MMSE), IADL (FAQ) and QoL (QoL‐AD) and decline on these measures over time. In line with previous work, we found several associations to baseline functional outcomes. Longitudinally, lower NPI‐hallucinations scores at baseline were associated to stronger increase of IADL dependency over time. Other clinical symptoms were not predictive of decline on MMSE, IADL or QoL over time. By contrast, several abnormalities on biological measures predisposed for decline on functional outcomes. Specifically, concomitant AD pathology (abnormal CSF pTau/Aβ42 ratio) was related to stronger decline on MMSE. Medial temporal and GCA were associated to steeper rate of decline in QoL. There were no significant associations of any of the determinants and change in FAQ.

**TABLE 3:** Baseline predictors of decline in cognition, IADL and quality of life (QoL)

	MMSE		FAQ		QoL‐AD	
	Baseline	Slope	Baseline	Slope	Baseline	Slope
Age	−0.07 ± 0.05	−0.00 ± 0.04	−0.03 ± 0.10	0.11 ± 0.05	0.14 ± 0.07*	−0.07 ± 0.03
Sex	−3.46 ± 0.79**	−1.12 ± 0.75	2.81 ± 1.75	0.55 ± 0.99	1.09 ± 1.21	−1.12 ± 0.62
Clinical symptoms						
MMSE	–	–	−0.98 ± 0.18**	−0.07 ± 0.12	0.2 ± 0.14	0 ± 0.08
Parkinsonism (UPDRS)	−0.06 ± 0.02*	−0.02 ± 0.02	0.16 ± 0.05**	−0.02 ± 0.03	−0.08 ± 0.04*	−0.01 ± 0.02
Hallucinations (NPI)	−0.19 ± 0.11	0.07 ± 0.09	0.52 ± 0.23**	−0.24 ± 0.11*	−0.33 ± 0.16*	0.13 ± 0.08
Fluctuations (MFQ)	−0.42 ± 0.25	0.06 ± 0.21	2.79 ± 0.49**	−0.38 ± 0.28	−1.09 ± 0.38**	0.2 ± 0.19
RBD (MSQ)	−0.34 ± 0.65	−0.22 ± 0.58	2.36 ± 1.39	−0.75 ± 0.75	−0.77 ± 0.99	0.55 ± 0.49
Depressive symptoms (GDS)	−0.27 ± 0.10**	−0.02 ± 0.09	0.73 ± 0.22**	−0.14 ± 0.11	−0.94 ± 0.14**	0.13 ± 0.08
Orthostatic hypotension (drop in SBP)	0.1 ± 0.56	−0.53 ± 0.47	0.03 ± 1.25	0.1 ± 0.63	−0.8 ± 0.87	0.24 ± 0.42
Biological measures						
CSF AD profile	−0.97 ± 0.65	−1.23 ± 0.54*	2.38 ± 1.49	1.17 ± 0.67	−0.35 ± 1.02	−0.04 ± 0.42
Abnormal EEG	−3.46 ± 1.64*	−0.03 ± 1.59	8.66 ± 3.81**	−1.75 ± 1.85	−7.6 ± 2.7**	1.65 ± 1.23
Abnormal DAT‐spect	−0.83 ± 92	0.05 ± 0.79	0.94 ± 2.08	0.39 ± 1.08	−0.22 ± 1.40	−0.84 ± 0.67
Medial temporal atrophy	−0.42 ± 0.41	−0.03 ± 0.31	0 ± 0.92	0.57 ± 0.40	0.68 ± 0.61	−0.81 ± 0.26**
Global cortical atrophy	−1.7 ± 0.51**	0.05 ± 0.42	2.3 ± 1.16*	0.48 ± 0.53	−0.95 ± 0.79	−0.73 ± 0.36*

*Note*: Data represent β ± SE. *ß* baseline = Association between predictor category and baseline outcome (MMSE, FAQ or QoL) *ß* Slope = association with annual decline, as estimated with interaction time*predictor with linear mixed models, corrected for age and sex. Bold depicts significant associations. *p < 0.05, **p < 0.5 after FDR correction.

Abbreviations: FAQ, Functional activities questionnaire; GDS, Geriatric depression scale; MFQ, Mayo Fluctuations Questionnaire; MMSE, Mini‐Mental State Examination; MSQ, Mayo Sleep Questionnaire; NPI, neuropsychiatric inventory; QoL‐AD, Quality of Life—Alzheimer's disease; SBP, systolic blood pressure; UPDRS, Unified Parkinson's disease rating scale.

## 4 DISCUSSION

In our prospective DLB cohort we observed worsening of symptoms and cognitive impairment, which was associated with increases in disease burden. Biomarkers of AD pathology and neurodegeneration at baseline were more predictive of the rate of decline of cognitive functioning, functional impairment and QoL than baseline clinical symptoms. Still, the degree of progression of clinical symptoms over time was related to progression in functional outcomes. Our findings have implications for therapeutic treatment strategies and give targets for the development of DLB specific outcome measures.

Congruent with previous studies, AD copathology was related to steeper cognitive decline.^33^, ^34^ Medial temporal and global atrophy were associated to decline in QoL. Atrophy scores could reflect staging of the underlying pathology and precede worsening of symptoms, more than just cognition, and therefore predispose for decline on outcomes that broadly measure progression such as QoL. Greater availability of biomarkers would enable more accurate prediction of disease progression. One counterintuitive finding was that patients who had lower severity of hallucinations showed steeper decline on IADL. Potentially, patients who had no or lower severity of hallucinations, had overall mild symptomatology at baseline and thus had more to lose over time. In line with this reasoning, progression of hallucinations was associated to changes in IADL and caregiver burden. So even though clinical symptoms at baseline were not as predictive for longitudinal functioning, a longitudinal relationship between symptom progression and functional outcomes is apparent. This finding underlines the importance of adequate symptomatic treatment, as this might reduce longitudinal disease burden.

As expected within a neurodegenerative disease, symptomatology and disease severity progressed over time. Global cognition declined in patients with DLB, with a decline of 2.1 points on MMSE per year, similar as found in previous studies in DLB^35^, ^36^ and in AD.^37^ When looking at individual cognitive domains, we found prominent decline on tests that address attention and processing speed. Most previous studies evaluating cognitive decline in DLB were limited by the use of screening tools that are particularly sensitive for memory impairment, while such tools might fail to capture decline in cognitive domains that are most affected in DLB.^38^ The trajectories of the MMSE might be an underrepresentation of the actual decline in cognitive functioning. Our findings underline the importance of carefully assessing all cognitive domains, especially attention. Impaired attentional processing is also a crucial feature involved in the core symptom fluctuations.^39^ Interestingly, although attention showed significant decline over time, the presence of fluctuations only mildly increased. It could be that the MFQ lacks sensitivity to capture changes in fluctuations, as it does not take into account fluctuation severity. On the other hand, increasing MFQ scores were associated to increases in IADL dependency and caregiver burden, suggesting that increasing presence of fluctuations impacts daily living of both patients and caregivers. This underlines the importance of carefully assessing fluctuations and where possible, provide adequate symptomatic treatment.

Or all core symptoms, parkinsonism and RBD were most likely to be present in the early stages of dementia, while hallucinations developed in a later stage for many of our patients. Progression of clinical symptoms was accompanied by changes in functional outcomes. Although we do not imply a causal relationship, it could be hypothesized that treatment of symptoms would have a beneficial effect on functional outcomes. Guidelines for treating DLB symptoms are available, yet knowledge on the effect of symptom treatment on IADL, QoL and caregiver burden is limited.^40^ Future RCTs could shed light on the causal relationship of our findings.

The main strength of this study is the prospective design with thorough and systematic annual clinical assessment. Secondly, this study estimated the trajectories not only in terms of cognitive decline but also incorporated QoL, IADL and caregiver burden over time, outcomes that patients and caregivers identified as relevant outcome measures.^41^ Few limitations should be noted. First, although follow‐up collection is still ongoing, already more than half of our patients was not able to complete the 4 year follow‐up duration, with an average time of 2 years between inclusion and loss‐to‐follow‐up. Patients who were lost to follow‐up were in more advanced disease stages at baseline, suggestive of attrition. While the loss to follow‐up is a limitation of the study, it also illustrative of the prognosis of DLB. Future studies with larger sample sizes could implement joint modelling, with both functional decline and mortality or nursing home admission included as outcomes. Second, by the observational nature of our cohort, several factors could have influenced progression that were not taken into account. For example, disease progression is likely influenced by the COVID‐19 pandemic and its associated lockdown measures. During the pandemic, social contacts and services such as daycare activities or physical therapy were severely restricted. DLB patients might be particularly vulnerable for the psychosocial effects of these restrictions, and it cannot be ruled out that restrictions have accelerated disease progression.^42^, ^43^ We paused the inclusion of patients during the pandemic, but we were able to continue our follow‐up measurements (with some delay). Patients were in varying stages of follow‐up during the pandemic, making this a difficult variable to take into account in analyses. A last limitation to be mentioned is that the findings are dependent on the measurements that were used, but it is not clear whether all measures are equally responsiveness to change. There is no consensus on how to measure DLB specific symptomatology and progression.^38^ A DLB‐specific, standardized set of outcomes would highly improve evaluation of disease progression and would enhance the potential of comparison with other studies.

To conclude, we presented the trajectories of cognitive domains and symptoms in our prospective DLB cohort. We found clinically relevant associations with disease burden. Biological markers could help in predicting which patients are at risk for worse prognosis. We propose that DLB specific outcome measures include a combination of cognitive measures, specifically tests for attention and processing speed, and functional measures, like IADL and QoL, as those capture clinically relevant aspects of the disease spectrum and are sensitive for decline.

## CONFLICT OF INTEREST

The authors have no conflicts of interest to declare.

## Supporting information

Supporting Information S1

## Data Availability

The data that support the findings of this study are available on request from the corresponding author. The data are not publicly available due to privacy or ethical restrictions.
