# Supplementary material for: Disease progression in dementia with Lewy bodies: A longitudinal study on clinical symptoms, quality of life and functional impairment
Source: Int J Geriatr Psychiatry. 2022 Nov 20;37(12):10.1002/gps.5839. doi: 10.1002/gps.5839 (PMC9828829; doi:10.1002/gps.5839)

| ***eTable 1:*** *Operationalization of core and suggestive symptoms and measures of disease burden* | | | |
| --- | --- | --- | --- |
| *Symptom* | *Scale / Questionnaire* | *Range* | *Cutoff* |
| Cognition | Neuropsychological assessment / MMSE | - | - |
| Parkinsonism | UPDRS-III (motor scale) | 0-108 | ≥1 on bradykinesia, with ≥1 on rigidity and/or ≥1 on resting tremor subscores |
| Visual hallucinations | NPI – Hallucinations subscale | 0-12 | ≥1 |
| Cognitive fluctuations | Mayo Fluctuations Questionnaire | 0-4 | ≥3 |
| RBD | Mayo Sleep Questionnaire | 0-4 | ≥1 |
| Orthostatic hypotension | Differences in blood pressure between supine and standing position | - | ≥20 mmHG drop in SBP or ≥10 mmHG drop in DBP |
| Depressive symptoms | Geriatric Depression Scale | 0-15 | ≥6 |
| IADL | Functional activities Questionnaire | 0-30 | - |
| Quality of life | Quality of Life – AD | 13-52 | - |
| Abbreviations: UPDRS: Unified Parkinson’s Disease Rating Scale, RBD: rapid eye movement behavior disorder, NPI: Neuropsychiatric Inventory, SBP: systolic blood pressure, DBP: diastolic blood pressure, IADL: instrumental activities of daily living | | | |

***eFigure 1****: Cluster dendrogram*


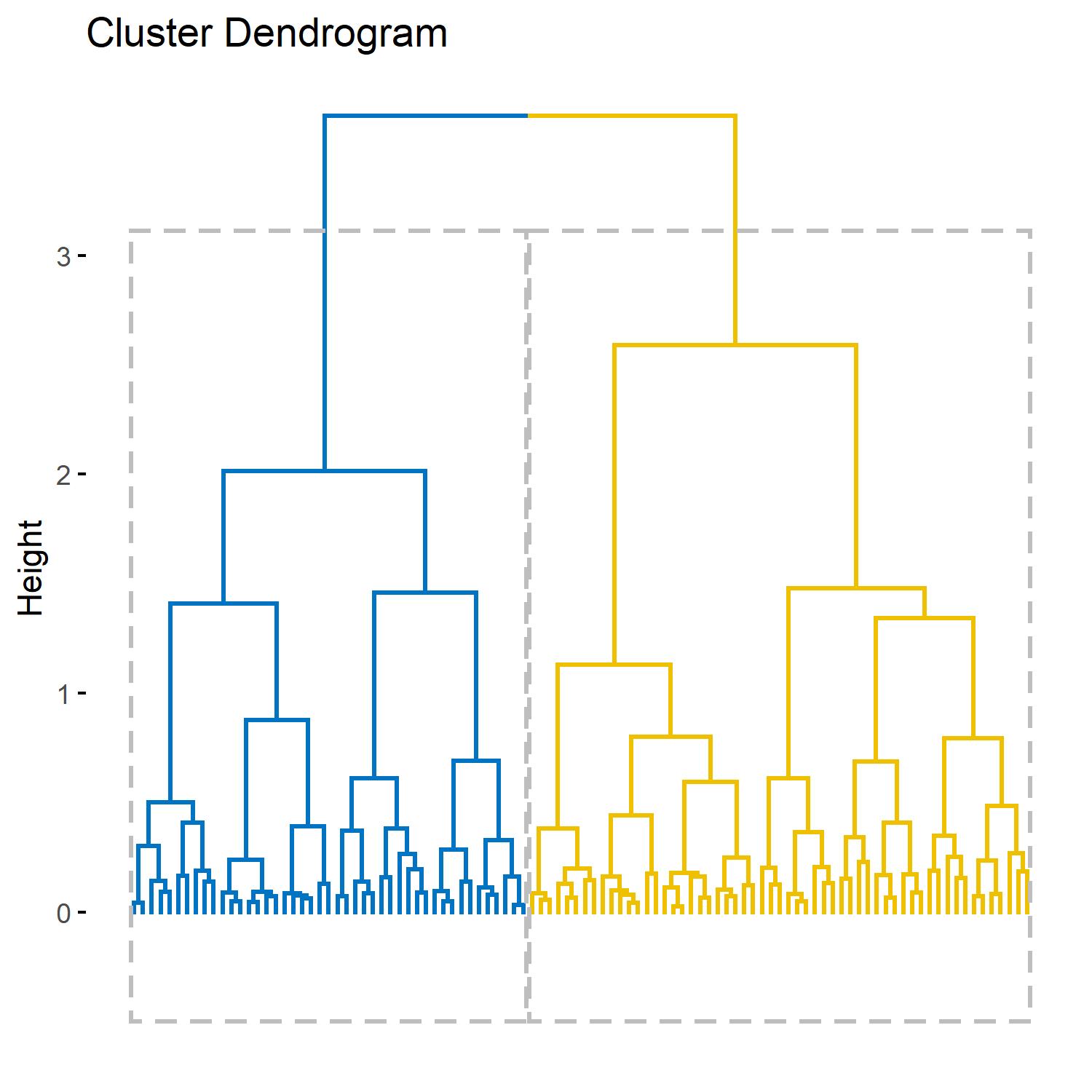

Supplement: Supplementary file 1 — Supporting Information S1 [file GPS-37-0-s001.docx]
